# Combined Adipose Tissue-Derived Mesenchymal Stem Cell Therapy and Rehabilitation in Experimental Stroke

**DOI:** 10.3389/fneur.2019.00235

**Published:** 2019-03-26

**Authors:** Jingwei Mu, Abdulhameed Bakreen, Miia Juntunen, Paula Korhonen, Ella Oinonen, Lili Cui, Mikko Myllyniemi, Shanshan Zhao, Susanna Miettinen, Jukka Jolkkonen

**Affiliations:** ^1^Department of Neurology, The People's Hospital of China Medical University, Shenyang, China; ^2^Department of Neurology, University of Eastern Finland, Kuopio, Finland; ^3^Faculty of Medicine and Health Technology, Tampere University, Tampere, Finland; ^4^Research, Development and Innovation Centre, Tampere University Hospital, Tampere, Finland; ^5^A. I. Virtanen Institute for Molecular Sciences, University of Eastern Finland, Kuopio, Finland; ^6^Neurocenter, Kuopio University Hospital, Kuopio, Finland

**Keywords:** stroke, cell therapy, rehabilitation, combination therapy, functional outcome, mechanisms, translational research

## Abstract

**Background/Objective:** Stroke is a leading global cause of adult disability. As the population ages as well as suffers co-morbidities, it is expected that the stroke burden will increase further. There are no established safe and effective restorative treatments to facilitate a good functional outcome in stroke patients. Cell-based therapies, which have a wide therapeutic window, might benefit a large percentage of patients, especially if combined with different restorative strategies. In this study, we tested whether the therapeutic effect of human adipose tissue-derived mesenchymal stem cells (ADMSCs) could be further enhanced by rehabilitation in an experimental model of stroke.

**Methods:** Focal cerebral ischemia was induced in adult male Sprague Dawley rats by permanently occluding the distal middle cerebral artery (MCAO). After the intravenous infusion of vehicle (*n* = 46) or ADMSCs (2 × 10^6^) either at 2 (*n* = 37) or 7 (*n* = 7) days after the operation, half of the animals were housed in an enriched environment mimicking rehabilitation. Subsequently, their behavioral recovery was assessed by a neurological score, and performance in the cylinder and sticky label tests during a 42-day behavioral follow-up. At the end of the follow-up, rats were perfused for histology to assess the extent of angiogenesis (RECA-1), gliosis (GFAP), and glial scar formation.

**Results:** No adverse effects were observed during the follow-up. Combined ADMSC therapy and rehabilitation improved forelimb use in the cylinder test in comparison to MCAO controls on post-operative days 21 and 42 (*P* < 0.01). In the sticky label test, ADMSCs and rehabilitation alone or together, significantly decreased the removal time as compared to MCAO controls on post-operative days 21 and 42. An early initiation of combined therapy seemed to be more effective. Infarct size, measured by MRI on post-operative days 1 and 43, did not differ between the experimental groups. Stereological counting revealed an ischemia-induced increase both in the density of blood vessels and the numbers of glial cells in the perilesional cortex, but there were no differences among MCAO groups. Glial scar volume was also similar in MCAO groups.

**Conclusion:** Early delivery of ADMSCs and combined rehabilitation enhanced behavioral recovery in an experimental stroke model. The mechanisms underlying these treatment effects remain unknown.

## Introduction

Stroke is one of the leading global causes of death and long-term disability, with about 5 million survivors becoming permanently disabled annually (1–3). Despite advances in acute stroke care (4), the narrow therapeutic time windows for early thrombolysis and thrombectomy make them available to only about 10% of stroke patients (5, 6). Safe and effective treatments beyond the acute phase are urgently needed.

Cell therapy represents a potential breakthrough in the treatment of stroke. In particular, mesenchymal stem cells (MSCs) are of major interest due to their advantages over other cell types, including their abundance and good availability (7), their relatively low immunogenicity (8) and tumorigenicity (9), and the lack of ethical concerns (10, 11). The non-invasive intravenous (IV) route has been most commonly used for delivery of MSCs in both preclinical and clinical studies (Cui et al. in press). More importantly, preclinical studies have revealed evidence for facilitation of behavioral recovery in animal models of stroke, e.g., improvements in sensorimotor functions (12–14).

Although still unclear, the putative mechanisms include secretion of neurotrophic factors that promote neuroprotection against inflammation (15), oxidative stress (14), and apoptosis (16). Neurorestorative mechanisms such as angiogenesis (17), neurogenesis (18), synaptogenesis (12), oligodendrogenesis (18), repair of white matter fiber tracts (19), and remodeling of neural circuits (20, 21) have also been proposed. In particular, local angiogenesis is required to provide sufficient oxygen and nutrients during cerebral reconstruction and remodeling of damaged tissue, thus this phenomenon plays an important role in the recovery of neural function after stroke (22). Indeed, it has been reported that a higher density of blood vessels resulted in reduced morbidity and prolonged survival of stroke patients (23, 24).

Recently, adipose tissue-derived mesenchymal stem cells (ADMSCs) have demonstrated their therapeutic potential in stroke models by improving the gross neurological condition (17, 18, 20, 25, 26), sensorimotor function (14, 15, 18, 19, 25, 27), as well as exerting beneficial effects on spatial learning and memory (7). In fact, the promising preclinical data laid the foundation for the first safety trial of ADMSCs in stroke patients (28). Unfortunately, the limited therapeutic efficacy in early patient studies have indicated that further preclinical studies are necessary in order to optimize the current cell treatment protocols.

After the acute phase, rehabilitation therapy is the only approved treatment for stroke survivors presenting with neurological deficits (29). In experimental settings, various rehabilitative approaches such as physical training (30, 31), skilled training (32–34), and special rehabilitative training devices (35, 36) have all been employed. In addition, housing the experimental animals in an enriched environment (EE) has also been used to provide multiple sensory, motor, social, and visual stimuli (37). Although very non-specific, housing in EE is one of the most promising approaches for improving an animal's sensorimotor functions after an experimental stroke (38, 39). EE has also been shown to improve spatial learning and memory in ischemia-reperfusion models (40).

The combination of different restorative approaches represents an intriguing approach to maximize treatment effects (41). Furthermore, cell-based therapies offer the possibility of combining different neurorestorative strategies to achieve an additive or even a synergistic therapeutic effect. However, only a few studies have been published (12, 13, 37, 42–45), and thus, more research is required in this regard to examine not only the stand-alone effects of each therapy, but also their potential combined effect (46, 47). Here, we hypothesized that the combination of an enriched environment with the IV infusion of ADMSCs after permanent middle cerebral artery occlusion (MCAO) would result in an improved behavioral recovery, perhaps even a maximal therapeutic effect. In order to explore the therapeutic window, we infused ADMSCs at either 2 or 7 d post-MCAO. Angiogenesis was evaluated as a possible repair mechanism related to treatment effect. In addition, glial cell staining was used to assess the extent of gliosis since the presence of a glial scar is considered to impede neuronal plasticity and prevent the functional recovery.

## Materials and Methods

### Animals

Ninety-seven adult male Sprague Dawley rats (Envigo, operation weight 274–340 g) were maintained in a controlled environment (temperature 20 ± 1°C; humidity 50–60%; light period 07:00–19:00) with free access to food and fresh water throughout the experiment. Animal care procedures were conducted according to the guidelines set by the European Community Council Directives 86/609/EEC; and this work was approved by the Animal Ethics Committee (Hämeenlinna, Finland).

### Preparation and Characterization of Human ADMSCs

The adipose tissue stem cell line RESSTORE01 (Master Cell Bank/Stock n°1—Donor RESSSTORE01, Batch n°: 591133643763) was cultured in the growth medium Alpha MEM (Gibco, Life technologies) supplemented with 5% human platelet lysate (Stemulate, Cook Medical, USA) and 1% Penicillin-Streptomycin (Lonza, Belgium). The medium was changed twice each week and cells were passaged when they reached 100% confluence. The cells were detached with TrypLE Select (Life Technologies™, Thermo Fisher Scientific) for 10 min at 37°C and then centrifuged at 1,000 rpm for 5 min. The RESSTORE01 cell phenotype was analyzed with flow cytometry (FACSAria Fusion Cell Sorter, BD Biosciences) at passage PX +1. Monoclonal antibodies against CD19-phycoerythrincyanine (PE-Cy7), CD45RO-allophycocyanin (APC), CD73-PE, CD90-APC (BD Biosciences), CD11a-APC, CD105-PE (R&D Systems Inc., Minneapolis, MN, USA), CD34-APC and HLA-DR-PE (Immunotools GmbH, Friesoythe, Germany) were used. The cells expressed (>95%) surface markers CD73, CD90, and CD105 and lacked the expression (<2%) of CD11a, CD19, CD34, CD45, and HLA-DR. Cells at passages PX +2/3 were used for the study.

### Permanent Middle Cerebral Artery Occlusion

Anesthesia was induced with 5% isoflurane in 30 O_2_/70% N_2_O and maintained during the operation with 2% isoflurane. The temperature of the rats was kept constant (37 ± 0.5°C) with a heating blanket and rectal probe (Harvard Homeothermic Blanket Control Unit; PanLab, Barcelona, Spain). In the occlusion of the right middle cerebral artery (MCA), the temporal muscle was removed to expose the temporal bone and a 2–3 mm diameter hole was drilled on top of the artery while cooling the bone with ice-cold 0.9% NaCl. The dura was carefully removed after which the artery was occluded with an electrocoagulator (Aesculap, Center Valley, PA, USA). Immediately after the MCA occlusion, both common carotid arteries (CCA) were occluded with micro-aneurysm clips for 60 min. After 1 h, the clamps were slowly released, the temporal muscle was replaced, and the incision/wound was sutured. The sham-operated rats went through all of the same procedures except for the occlusion of MCA and CCAs. To assist rehydration, 5 ml of 0.9% NaCl was given intraperitoneally. Buprenorfine (Temgesic, 0.03 mg/kg) was injected subcutaneously immediately after surgery as an analgesic.

### Magnetic Resonance Imaging

**Figure 1:**
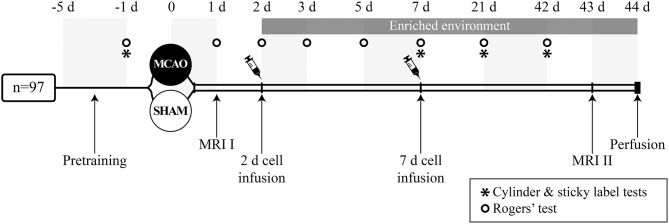
Study design.

Magnetic resonance imaging (MRI) was performed 24 h after the operation and on post-operative day 43 (Figure 1) using a Bruker 9.4 T horizontal scanner. The rats were anesthetized with 5% isoflurane in 30 O_2_/70% N_2_. After induction, the anesthesia was maintained throughout the imaging with 1.5% isoflurane inhaled through a nose mask. In the determination of the infarct volume, T2 weighted multi-slice images were acquired using a RARE sequence with the following parameters: time-to-repetition TR = 2.5 s, effective time-to-echo effTE = 40 ms, RARE factor 8, matrix size of 256 × 256, field-of-view of 30 × 30 mm, 15 slices with a slice thickness of 1 mm. ${\text{T}}_{2}^{*}$ weighted images were obtained using a standard gradient echo imaging sequence from the same slices with identical resolution and TR = 700 ms, TE = 15 ms, flip angle ~50°. The cortical infarct volume was measured using in-house written Matlab software. Animals with infarct size <20 mm^3^ (*n* = 5) or >150 mm^3^ (*n* = 2) were excluded from the data analysis. These exclusion criteria had been decided before the experiment.

### Cell Treatment and Housing in Enriched Environment

The animals were sequentially assigned to experimental groups based on initial screening on MRI to ensure that the infarct size did not differ between experimental groups before treatment (Table 1). Two days after the occlusion procedure, isoflurane anesthetized rats were slowly infused with 2 million cells/1 ml 0.9% NaCl into the tail vein. Vehicle groups were treated with 1 ml 0.9% NaCl. Additional animals were treated 7 days after MCAO (Figure 1). Body weight was recorded during the follow-up as part of the safety assessment. After the infusion of the cells, half of the rats were moved to an enriched environment that consisted of two large metal cages (61 × 46 × 46 cm) that were connected by a tunnel. The cages contained ladders, tunnels, shelves and a running wheel to provide sensorimotor stimuli. Novel objects (e.g., toys, wooden balls) were changed every second day. Altogether, 8–9 animals were housed per cage. The animals in the non-rehabilitation group were housed in groups of three rats in standard cages (53 × 32.5 × 20 cm).

**Table 1:** Experimental groups.

	**Groups**	**Treatment**		**Housing**		**Timing**	
		**Vehicle**	**Cell**	**Standard**	**EE**	**2 d**	**7 d**
SHAM	SHAM+V+S (*n* = 8)	x		x		x	
	SHAM+C+S (*n* = 8)		x	x		x	
	SHAM+V+EE (*n* = 8)	x			x	x	
	SHAM+C+EE (*n* = 8)		x		x	x	
MCAO	MCAO+V+S (*n* = 12)	x		x		x	
	MCAO+C+S (*n* = 10)		x	x		x	
	MCAO+V+EE (*n* = 10)	x			x	x	
	MCAO+C+EE (*n* = 11)		x		x	x	
	MCAO+V7+EE (*n* = 8)	x			x		x
	MCAO+C7+EE (*n* = 7)		x		x		x

*SHAM, sham-operated; MCAO, middle cerebral artery occlusion; V, 2 d vehicle; C, 2 d cell infusion; V7, 7 day vehicle; C7, 7 day cell infusion; S, standard housing; EE, enriched environment*.

### Behavioral Testing

All behavioral tests were carried out in a blinded manner 1 day before the occlusion procedure and on post-operative days (between 9 and 12 a.m.) as shown in Figure 1. Behavioral impairment was assessed using the Rogers', cylinder and sticky label tests. Two animals were excluded from the sticky label test analysis due to problems with their teeth and behavioral peculiarities.

#### Rogers' Test

The Rogers' functional evaluation scale was used to assess the gross behavioral impairment, including reflexes, sensory responses, and simple motor functions (17). It consists of a 7-point behavioral rating scale: score 0—no functional deficit; score 1—failure to extend left forepaw fully; score 2—decreased grip of the left forelimb while tail gently pulled; score 3—spontaneous movement in all directions, contralateral circling only if pulled by the tail; score 4—circling or walking to the left; score 5—walking only when stimulated; score 6—unresponsive to stimulation with a depressed level of consciousness; and score 7—dead.

#### Cylinder Test

The cylinder test was used to measure spontaneous forelimb use and imbalance between the non-impaired and impaired forelimbs (48). In this test, the rat was placed in a transparent plastic cylinder (Ø 20 cm) and video-recorded (5 min) through a mirror placed under the cylinder. The videotaped exploratory activity in the cylinder was analyzed for 1 to 3 min using a program with slow motion capabilities. The number of contacts on cylinder by either the impaired or the non-impaired forelimb or both forelimbs was counted (minimum 30 contacts). The imbalance in forelimb use was calculated as: [(use of impaired forelimb + 0.5 × use of both forelimbs) ÷ (total contacts)] × 100%.

#### Sticky Label Test

The sticky label test was used to evaluate sensory function and motor learning, and was performed as previously described (48). Before testing, the animals were familiarized with handling and the testing cage. In the test, a white colored circular label (Ø 9 mm, Tough-Spots, Diversified Biotech) was placed on the distal-radial region of both wrists and rat was moved to a test cage. The time for the first contact to the label and time to remove the label were measured. A maximum time of 120 s was set if the rat was not able to contact or remove the label.

### Histology

After the behavioral assessment, rats were perfused on post-operative day 44 (Figure 1) with 0.9% NaCl followed by 4% paraformaldehyde in 0.1 M phosphate buffer, pH 7.4. The brains were carefully removed from the skull, post-fixed, and cryoprotected. Brain sections (35 um) were cut using a sliding microtome and stored in antifreeze solutions at −20°C. Systematically sampled sections with a random start covering the entire infarct were selected for staining and analysis. One series of sections was stained with the angiogenesis marker, anti-RECA-1 antibody, and another series with the gliosis marker, anti-GFAP antibody.

#### RECA-1 Immunohistochemistry

The sampled sections were stained with anti-RECA-1 antibody to visualize blood vessels. Briefly, free-floating sections were washed in 0.1 M phosphate buffer (PB) (3 × 15 min) and kept overnight in a cold room. They were then rinsed with 0.5 M Tris buffered saline + Triton (TBS-T) at pH 7.6 (3 × 5 min) before treatment with the primary antibody mouse anti-rat RECA-1 (AbD Serotec, Bio-Rad) at 1:2000 in TBS-T at pH 7.6 for 18 h on a shaker table in the dark at room temperature. Afterwards, the sections were rinsed in TBS-T at pH 7.6 (3 × 5 min) and then treated with secondary antibody biotinylated anti-mouse IgG (Vector) made in goat at 1:500 in TBS-T at pH 7.6 for 2 h on a shaker table, room temperature. Subsequently, the sections were rinsed in TBS-T at pH 7.6 (3 × 5 min), followed by treatment with Streptavidin-horseradish peroxidase conjugate (GE Healthcare UK Limited) at 1:1000 in TBS-T at pH 7.6 for 2 h on a shaker table at room temperature. The sections were again rinsed in TBS-T at pH 7.6 (3 × 5 min) and then carefully developed with filtered nickel-intensified DAB (Sigma) for ~3 min. Excess DAB was rinsed with PB (3 × 4 min) before the sections were mounted and kept at 37°C overnight. The mounted sections were then washed in PB for 5 min before they were counterstained with thionin to reveal the neuroanatomy and delineate the perilesional area. They were then cleared in xylene (2 × 5 min) and coverslips were mounted using Depex.

#### GFAP Immunohistochemistry

Other sets of sampled sections were stained with anti-GFAP antibody to visualize astrocytes. Briefly, free-floating sections were washed in PB (3 × 15 min) and kept overnight in a cold room. They were then washed in TBS-T at pH 8.6 (2 × 5 min) before incubating in primary antibody mouse anti-GFAP (Sigma) at 1:1000 in TBS-T at pH 8.6 for 18 h on a shaker table in the dark at room temperature. The sections were again rinsed in TBS-T at pH 8.6 (3 × 5 min) and then treated with secondary antibody goat anti-mouse IgG-HRP conjugated (Invitrogen) at 1:500 in TBS-T at pH 8.6 for 2 h on a shaker table at room temperature. Afterwards, the sections were rinsed in TBS-T at pH 8.6 (3 × 5 min) and then carefully developed with filtered nickel-intensified DAB for ~3 min. Excess DAB was rinsed with PBS (3 × 4 min) before the sections were mounted and kept at 37°C overnight. Finally, the mounted sections were cleared in xylene (2 × 5 min) and coverslips were mounted using Depex.

### Histological Analysis

The analysis was done with the aid of Stereo Investigator software (MicroBrightField, Inc., VT, USA) attached to an ECLIPSE E600 microscope (Nikon, Japan) via a 3-Chip CCD color video camera (QImaging, Canada). A motorized stage with a microcator (Heidenhain EXE 610C) attachment (providing a 0.1 μm resolution in the Z axis) was mounted on the microscope.

In the assessment of angiogenesis, the perilesional tissue 200 μm from the ischemic border around the lesion was first outlined under 4x magnification (N.A. 0.06) and thereafter blood vessels were counted under 20x magnification (N.A. 0.75). To determine the vessel density in sections stained with RECA-1, we used the virtual sphere method (49). A three-dimensional sampling hemisphere (“space ball”) with a radius of 20 μm was placed within a sampling box with known dimensions (*x* = 200 μm, *y* = 200 μm, and *z* = 20 μm) and focused through the section thickness. The x-y steps giving the distance between sampling areas was 400 μm (x-axis) by 400 μm (y-axis), aimed at generating counts of about 300 vessel intersections per animal. In order to calculate the total length of blood vessels, the following equation was used: L_total_ = ΣQ × 2 × 1/ssf × 1/asf × 1/tsf × [v/a], where Q is the number of intersections between vessels and the probe, ssf (section sampling fraction) is 1/15, asf (area sampling fraction) is 0.03, tsf (tissue sampling fraction) is 1, and v/a is 19.2 μm [defined as the ratio of the volume (v) of the counting frame (sampling box) to the surface area (a) of the hemisphere probe (space ball)]. The vessel density was counted as the ratio of measured length and the total volume of perilesional area.

The optical fractionator technique was used to measure the glial scar and to assess the total number of GFAP labeled cells in the perilesional area (50). The glial scar was defined as glial cell aggregation. The perilesional zone was defined as a 200 μm wide cortical zone directly surrounding the scar. The cut thickness of the tissue was 35 μm and the average mounted thickness was 20 μm. The size of the counting frame was 100 × 100 μm, with the height of the dissector cube of 20 μm and a grid of 200 × 200 μm. The perilesional area and the scar area were traced using a 2x objective (N.A. 0.10) and the number of GFAP positive cells was counted using a 20x objective (N.A. 0.75). A GFAP positive cell was counted when the cell soma did not intersect with the uppermost focal plane (exclusion plane) and the lateral exclusion boundaries of the counting frame. The perilesional area reference volume was determined by adding the traced perilesional area for each section multiplied by the distance between sections sampled. For the total number of GFAP positive cells, the following equation was used: N_total_ = ΣQ × 2 × 1/ssf × 1/asf × 1/tsf. The number of GFAP labeled cells was then related to a total perilesional volume. The enclosed volume of the scar was acquired from the contour summary provided by Neurolucida software (MicroBrightField, Inc.).

### Statistical Analysis

Statistical analyses were performed using SPSS software for Windows (version 25). One-way analysis of variance (ANOVA), followed by the LSD *post-hoc* test if necessary, was used to analyze the statistical differences between groups in infarct volume, RECA-1, and GFAP staining. The Kruskal-Wallis H test, followed by the Mann-Whitney *U*-test if necessary, was used to compare the neurological scores. Repeated measures ANOVA, followed by the LSD *post-hoc* test if necessary, was used to analyze behavioral data from the cylinder and sticky label tests. Spearman and Pearson correlations were used to examine the relationship between behavioral impairment and infarct size, angiogenesis, and gliosis. Data are expressed as mean ± standard deviation (SD).

## Results

### Intravenous ADMSC Infusion Was Not Associated With Mortality or Adverse Effects

To assess the safety of ADMSC treatment, we carefully monitored the rats during the follow-up. There was no mortality after infusion of either cells or vehicle. Weight gain was similar in all experimental groups (see Supplemental Table 1). Safety was also evaluated using the Rogers' scale on post-operative days 1, 2, 3, 5, 7, 21, and 42 (see Supplemental Table 2). There was a statistically significant difference (*P* < 0.001) in neurological scores at all time points after the occlusion procedure, demonstrated by the better performance in the sham-operated groups compared to the MCAO groups. No statistically significant differences were found between vehicle- and cell-treated groups at all post-operative time points (data not shown).

### Infarct Size Was Not Affected by ADMSCs

**Figure 2:**
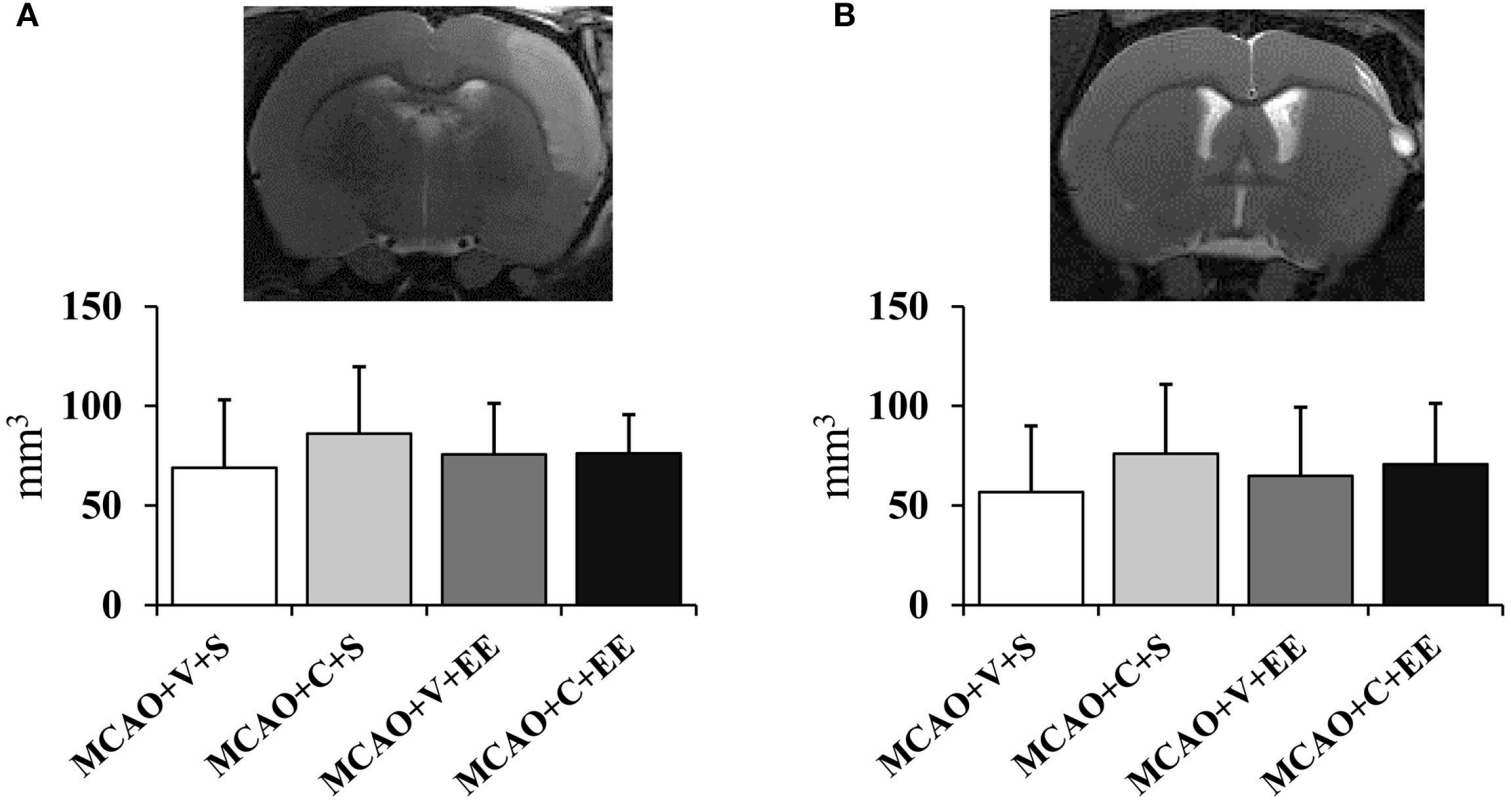
Infarct size and location. Infarct size 24 h **(A)** after MCAO in rats and at the end of the follow-up on day 43 **(B)**. MRI images show the location of a typical infarct in the sensorimotor cortex at these time points.

There were no differences in the cortical infarct size between the experimental groups at 24 h after operation (Figure 2A). In order to determine whether the therapeutic effect would be related to delayed neuroprotection, the infarct size was measured also at the end of the follow-up (Figure 2B). A variable maturation of infarct was observed, in many cases leading to a liquid-filled cyst. However, the infarct size did not differ between control and cell-treated groups on post-operative day 43.

### ADMSC Infusion and EE Improved Spontaneous Forelimb use in MCAO Rats

Treatment effects were assessed by the performance of the animals in the cylinder and sticky label tests on post-operative days 7, 21, and 42.

#### Cylinder Test

**Figure 3:**
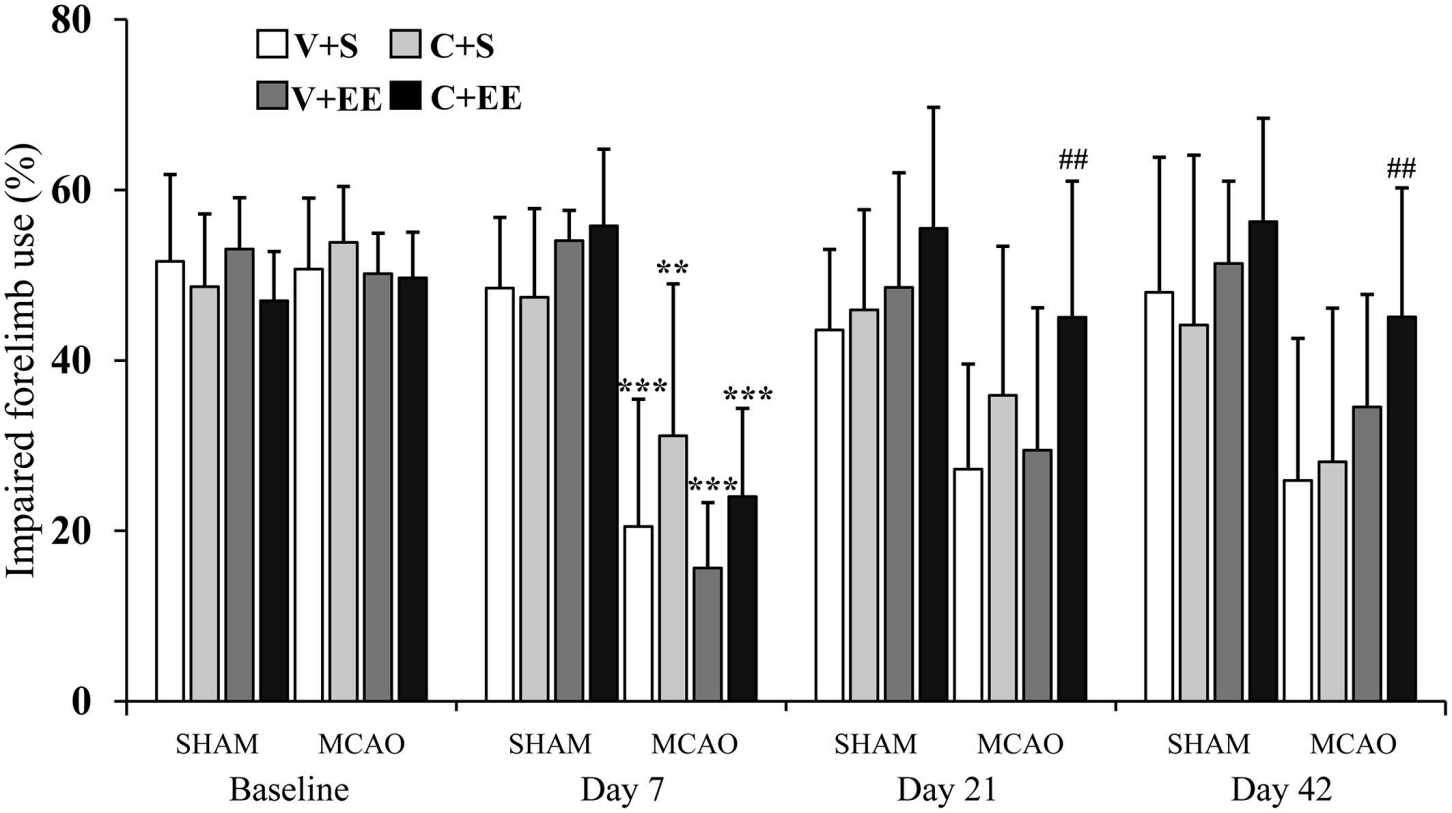
Cylinder test. Impaired forelimb use during the vertical exploration in the cylinder test was improved by ADMSC treatment and housing in an enriched environment in MCAO rats. Statistical significance: ^**^P < 0.01; ^***^P < 0.001 (compared to SHAM+V+S), ##*P* < 0.01 (compared to MCAO+V+S). SHAM, sham-operated; MCAO, middle cerebral artery occlusion; V, 2 d vehicle; C, 2 d cell infusion; S, standard housing; EE, enriched environment.

The imbalance in the spontaneous forelimb use during vertical exploration was assessed in the cylinder test (Figure 3). There was a significant overall group effect (*P* < 0.001) and time × group interaction (*P* < 0.001). In the more detailed analysis, significant time × group interactions were found between standard-housed vs. EE-housed (*P* < 0.01) as well as with vehicle-treated vs. ADMSCs-treated (*P* < 0.05) animals, showing that recovery of the impaired forelimb was different between the groups. All MCAO groups were different from SHAM+V+S on post-operative day 7. The MCAO+V+S group displayed a slower recovery on post-operative day 21. MCAO+V+S control rats were different from the MCAO+C+EE group on post-operative days 21 and 42 (*P* < 0.01). The amount of spontaneous forelimb use at the end of the follow-up correlated with infarct size on post-operative day 1 (*r* = −0.440, *P* < 0.01), blood vessel density (*r* = −0.278; *P* < 0.01), and number of glial cells (*r* = −0.550; *P* < 0.001) (Table 2).

**Table 2:** Correlations of behavioral outcome at the end of the follow-up with infarct size (24 h), angiogenesis and gliosis.

	**Rogers' test**	**Cylinder test**	**Sticky label test**	
			**Time to first contact**	**Time to remove**
Infarct size (24 h)	*r* = 0.311; *P* < 0.05	*r* = −0.440; *P* < 0.01	NS	NS
Blood vessel density	NS	*r* = −0.278; *P* < 0.01	NS	NS
Number of glial cells	*r* = 0.530; *P* < 0.001	*r* = −0.550; *P* < 0.001	NS	NS
Glial scar volume	NS	NS	NS	NS

*NS, not significant*.

#### Sticky Label Test

**Figure 4:**
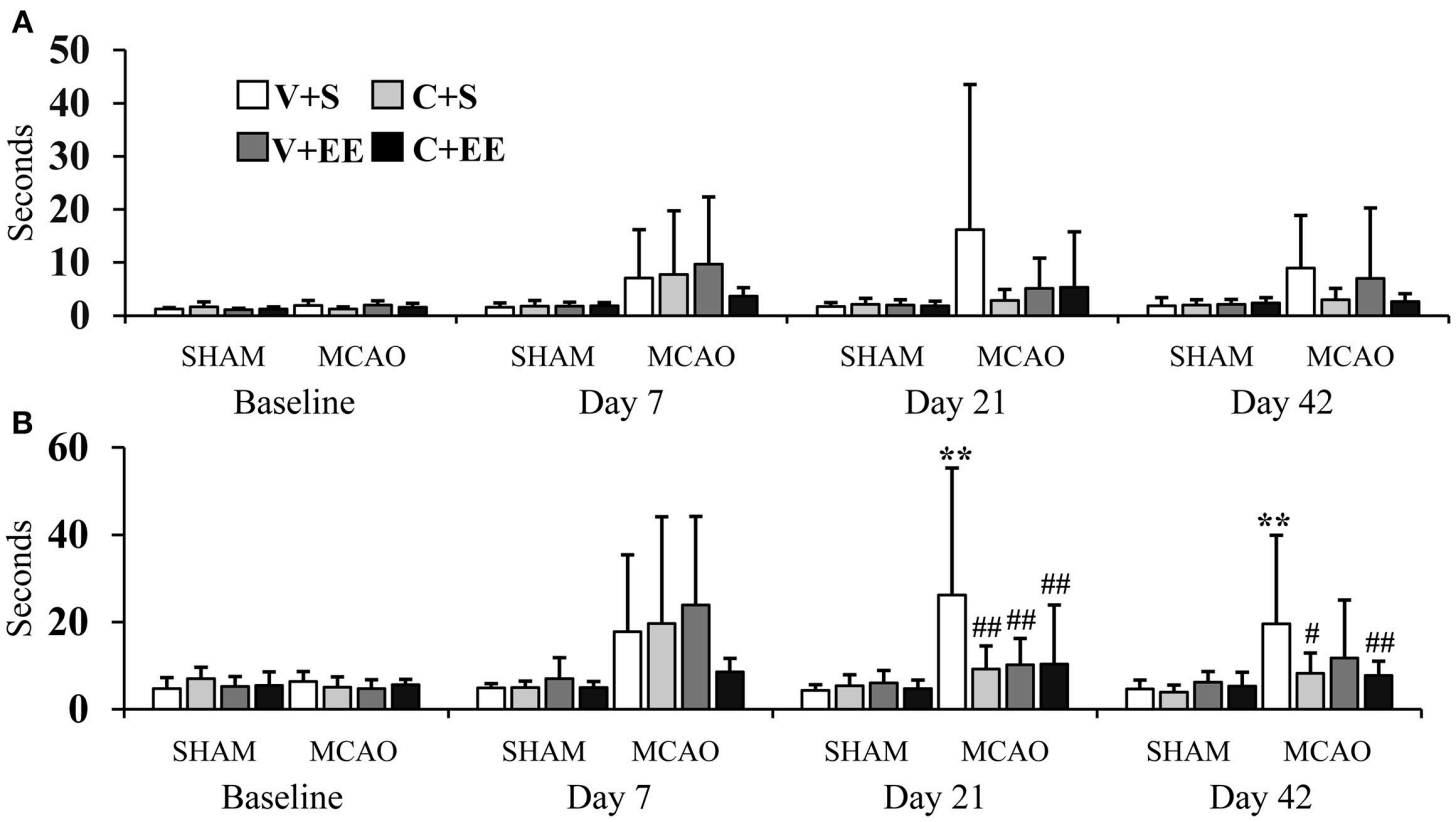
Sticky label test. Time needed before the first contact was not different between the experimental groups **(A)**. The time to removal of sticky label from impaired forelimb was increased in MCAO rats, an effect partially reversed by ADMSCs an EE **(B)**. Statistical significance: ^**^P < 0.01 (compared to SHAM+V+S); #*P* < 0.05, ##*P* < 0.01 (compared to MCAO+V+S). SHAM, sham-operated; MCAO, middle cerebral artery occlusion; V, 2 d vehicle; C, 2 d cell infusion; S, standard housing; EE, enriched environment.

The sticky label test was performed to evaluate sensorimotor function and motor learning (Figure 4). With respect to the impaired forelimb, there were no significant overall group effect (*P* = 0.055) or any time × group interaction (*P* = 0.138) on the time for the first contact with the label (Figure 4A). With respect to the time to remove the label, there was a significant overall group effect (*P* < 0.01) and a time × group interaction (*P* < 0.01). MCAO+V+S rats used more time to remove the label as compared to SHAM+V+S (*P* < 0.01) or animals in the other MCAO groups (*P* < 0.01) on post-operative day 21. At the end of the behavioral follow-up, MCAO+V+S controls were different from SHAM+V+S (*P* < 0.01), MCAO+C+S (*P* < 0.05), and MCAO+C+EE (*P* < 0.01) groups (Figure 4B). With respect to the non-impaired forelimb, there were no significant overall group effects and time × group interactions for the time to the first contact with the label or for the time required to remove the label (data not shown).

### Cell Infusion on Post-operative Day 7 Was Not Effective as When Cells Infused on Day 2

**Figure 5:**
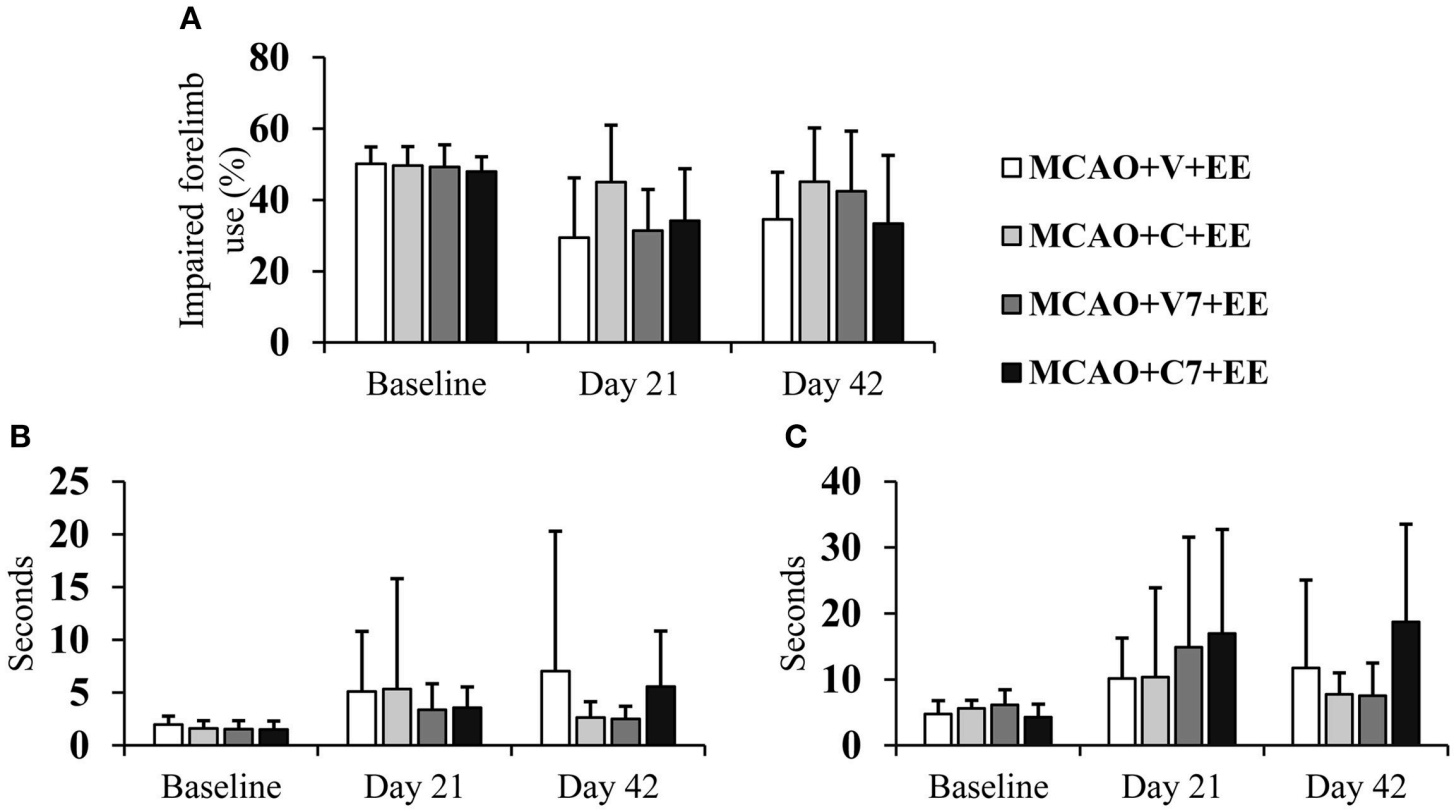
Timing of cell delivery. Early ADMSC delivery (48 h) in MCAO rats seemed to improve forelimb use when compared to delayed delivery (7 d) as assessed in the cylinder test **(A)** and sticky label test: time to touch **(B)**, time to remove **(C)**. MCAO, middle cerebral artery occlusion; V, 2 d vehicle; C, 2 d cell infusion; V7, 7 day vehicle; C7, 7 day cell infusion; EE, enriched environment.

The optimal therapeutic time window for combined therapy was assessed by comparing treatment starting on days 2 and 7. There were no significant overall group effects or time × group interactions in the cylinder test (Figure 5A) or sticky label test (Figures 5B,C), when cell infusion on days 2 and 7 were compared. However, a trend toward a better recovery was observed when cells were delivered earlier.

### The Extent of Angiogenesis or Gliosis Was Not Related to the Behavioral Recovery

Perilesional cortex undergoes a major reorganization after cerebral ischemia, a phenomenon thought to be related to the behavioral recovery. Here, we measured the formation of new blood vessels as a mechanism behind the behavioral recovery and we also assessed the extent of glial scar formation that might hinder the recovery process.

#### Angiogenesis

**Figure 6:**
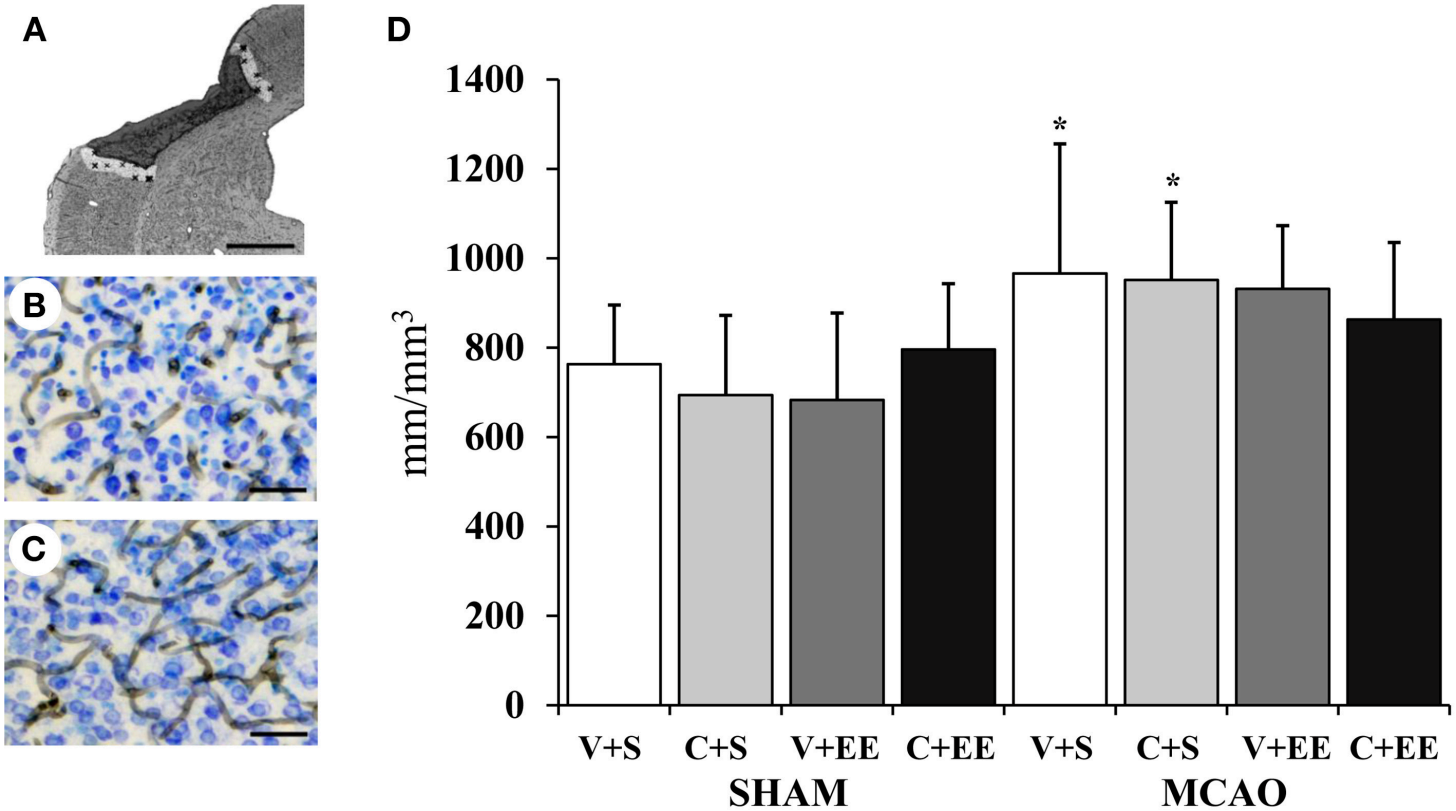
Quantification of perilesional angiogenesis. Definition of perilesional cortex 200 μm from the border of ischemic core **(A)**. Representative RECA-1 staining with thionin counterstaining in sham-operated **(B)**, and MCAO **(C)** rats. Stereological analysis showed ischemia-induced increase in blood vessel density in perilesional cortex **(D)**. Scale 1 mm **(A)**, 50 μm **(B,C)**. Statistical significance: ^*^P < 0.05 (compared to SHAM+V+S). SHAM, sham-operated; MCAO, middle cerebral artery occlusion; V, 2 d vehicle; C, 2 d cell infusion; S, standard housing; EE, enriched environment.

The overall distribution of RECA-1 stained blood vessels in the cortex is shown for a sham-operated rat (Figure 6B) and for a MCAO rat (Figure 6C). Figure 6A shows how the perilesional cortex was defined. There was a significant difference (*P* < 0.01) in the blood vessel density in the cortex due to the difference between sham-operated and MCAO animals. However, there was no significant difference between the MCAO groups (Figure 6D).

#### Glial Cells and Glial Scar

**Figure 7:**
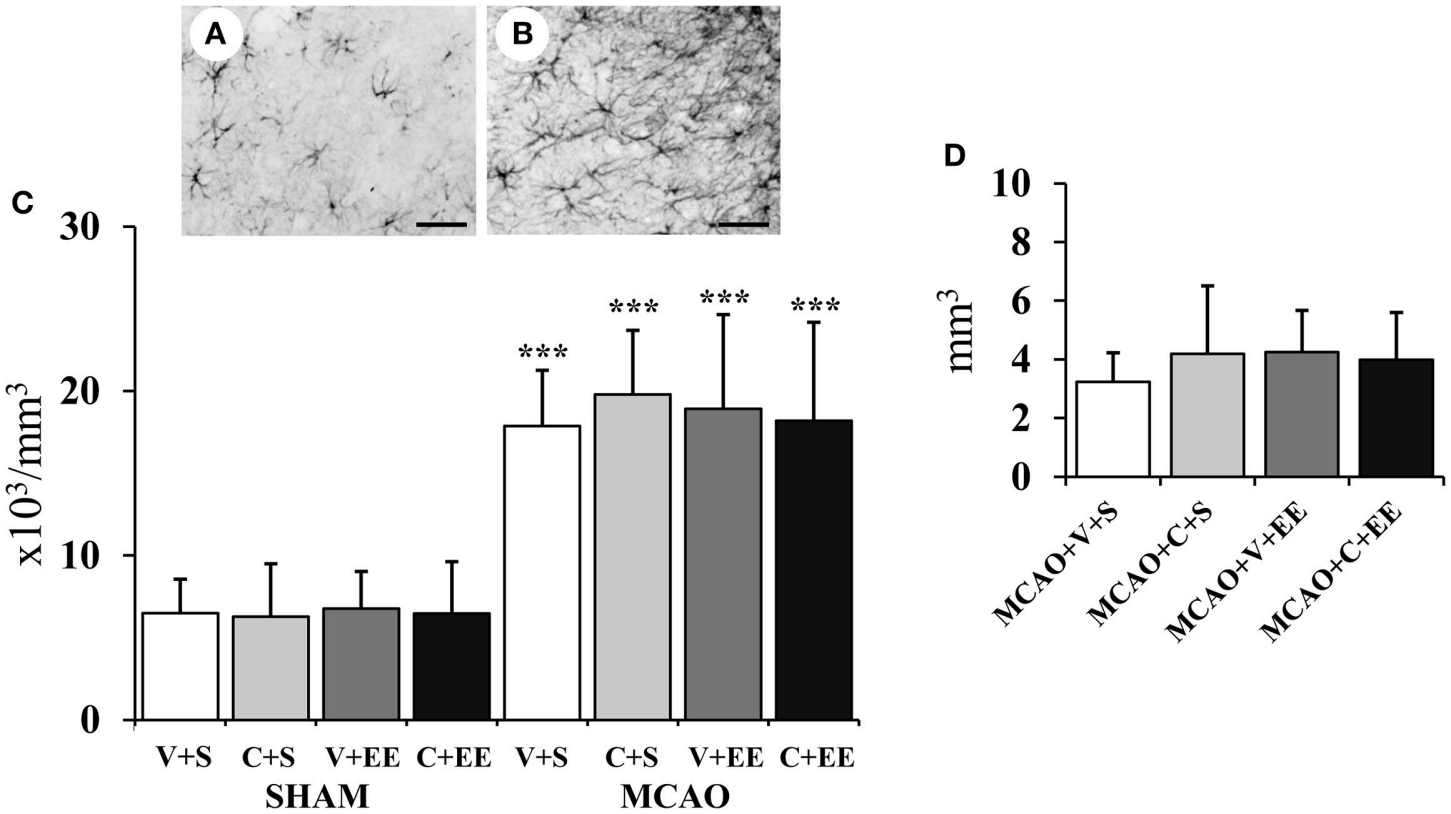
Quantification of perilesional gliosis. Representative staining of GFAP-stained sections from a sham-operated rat **(A)** and an MCAO rat **(B)**. Stereological analysis revealed an ischemia-induced increase in the number of glial cells in the perilesional cortex **(C)**. Glial scar volume did not differ between the MCAO groups **(D)**. Scale 50 μm. Statistical significance: ^***^P < 0.001 (compared to SHAM+V+S). SHAM, sham-operated; MCAO, middle cerebral artery occlusion; V, 2 d vehicle; C, 2 d cell infusion; S, standard housing; EE, enriched environment.

Figure 7A shows GFAP staining for resting glial cells in a sham-operated rat with Figure 7B revealing the change in the phenotype into hypertrophic reactive astrocytes and scar-forming astrocytes in the perilesional cortex after ischemia. There was a significant overall effect in the number of glial cells in perilesional cortex (*P* < 0.001) (Figure 7C); this was due to an increase in the number of glial cells in MCAO animals. However, there was no significant difference between the MCAO groups nor was there any significant difference between MCAO groups in the glial scar volume (Figure 7D).

## Discussion

In this experimental model of stroke, we investigated whether the therapeutic effect of human ADMSCs could be further enhanced by rehabilitation. We found that cell therapy or rehabilitation alone improved the functional recovery of the impaired forelimb, when treatment was started 2 days after the induction of ischemia. Combined therapy further improved the behavioral outcome. Delayed neuroprotection, angiogenesis or altered gliosis did not explain the behavioral improvement.

### Methodological Issues

ADMSCs are easy to obtain in large quantities (7), have no ethical concerns (10, 11), and have an excellent safety profile (9, 17). The same cell product is being used in the ongoing RESSTORE clinical trial (NCT03570450). The MCAO stroke model was selected according to recent SRRR guidelines (51). It produces a consistent infarct in the sensorimotor cortex causing a moderate behavioral impairment and partial spontaneous recovery in the long-term follow-up. The somewhat large variability in infarct size, which was also reflected in behavioral scores, and thus complicating the statistical analysis, is possibly due to the rat strain. The selected outcome measures are sensitive at identifying treatment effects and are not affected by repeated testing, but combined treatment effects can be missed by the presence of spontaneous recovery and of a ceiling effect.

Our study also has some methodological limitations. Young male rats were used, although the main unmodifiable risk factor for stroke is aging, which may impair brain repair including angiogenesis (52), and also decrease the therapeutic effect of cells (53). Thus, aging as well as co-morbidities such as hypertension and diabetes (54) should be addressed despite the fact that they further complicate the study design. As far as we are aware, this is the first time that stereology has been used to evaluate the extent of brain repair in a stroke model. However, since the RECA-1 antibody stains all blood vessels, perhaps CD31 (PECAM-1) staining would have revealed post-stroke angiogenesis (52). The only reliable way to differentiate between new and old blood vessels would be to conduct BrdU/RECA-1 double staining.

### Intravenous ADMSC Delivery Is Safe

The safety of new therapies is of the utmost importance (55); however, it is often overlooked unless demanded by the regulatory authorities. In the present study, we did not observe mortality due to pulmonary embolism or any other reasons; weight gain was similar in all groups; the gross neurological evaluation revealed no abnormalities in sham-operated or ischemic rats after cell treatment; and MRI images were clean with no sign of bleeding. These all support the concept that the used cell product, dose and delivery route were safe in our rat stroke model. However, regulatory safety studies will need to be carried out before embarking on patient trials.

### Early ADMSC Transplantation Improves Sensorimotor Recovery

Promising behavioral improvements have been reported in experimental stroke models following IV infusion of MSCs (12, 13), including ADMSCs (15, 19, 20, 27). Consistent with these studies, we observed that the spontaneous use of the impaired forelimb in the cylinder test had been improved by the infusion of the ADMSCs. In addition, 2 d post-stroke delivery of ADMSCs to the MCAO rats resulted in a significant decrease in the time needed to contact and particularly, to remove sticky labels from the impaired forelimb.

The critical time window during which the brain is most responsive to cell therapy is not known. In most of the experimental studies, cells have been infused within 24 h after brain ischemia (Cui et al. in press), although later time points have also been evaluated (7, 56). Here we found a trend toward a better recovery when the cells were delivered earlier. While early delivery might provide neuroprotection, that hypothesis was however not supported by the present data. Furthermore, the recent phase II MASTERS study in stroke patients supports the efficacy of early delivery (57). In addition, there is accumulating evidence suggesting that intravenously delivered cells modulate peripheral immune systems within another specific time window i.e., 24–48 h post-stroke, improving functional recovery (58). This is the target in the ongoing phase III MASTERS-2 study in stroke patients (NCT03545607).

The relatively modest behavioral improvement in our study could be due to the loss of cells after xenogenic transplantation. The differences in the clearances of human and rat bone marrow-derived MSCs after systemic infusion support this proposal (59). However, both allogenic and xenogenic delivery of ADMSCs have been effective in experimental stroke (7, 15, 17, 26, 27, 56). Cell entrapment in lungs might also affect the efficacy of the treatment, although it is still not clear whether the cells have to enter the brain parenchyma to play a role in behavioral recovery (60). Lastly, when robust study quality criteria are applied (e.g., randomization, blind assessment), the preclinical results are not so convincing and are more in line with the relatively modest evidence of efficacy emerging from small patient studies.

### EE Results in a Modest Improvement in Sensorimotor Functions

Experimental stroke rehabilitation is an emerging research area. The available data does not support the belief that one particular approach is superior to the others (61). In our study, we examined the benefits of an enriched environment, which provides spatial, sensory, motor, and social stimuli for rodents. This is known to be one of the most powerful forms of experimental rehabilitation (39), improving not only gross neurological and sensorimotor functions (38, 62–64), but also spatial learning and memory after cerebral ischemia (40, 65). In our study, housing in the enriched environment, when started on post-operative day 2, improved spontaneous forelimb use in the cylinder test and reduced the removal time in the sticky label test.

A major problem with enriched environment is that the overall stimulation varies and is dependent on the activity of the animal. Thus, it remains to be determined whether intensive or forced physical training or a more controllable and task-specific exercise such as skilled forelimb reaching to supplement the enriched environment would have been more effective (66). One way to increase treatment contrast would be to house control rats in single cages. In the present study, three rats were housed in the same cage as demanded by the animal ethics committee.

### Combined Therapy Seems to Further Improve Sensorimotor Functions

The wide therapeutic time windows for both cell therapy and rehabilitation allow their combination. Although the prospect of combining different neurorestorative approaches to maximize the therapeutic effect is theoretically very interesting (41), to date, very few studies have utilized this research strategy (12, 13, 37, 42–45). In our study, we found that combining ADMSC at 2 d cell delivery with EE in MCAO animals increased the spontaneous use of the impaired forelimb during vertical exploration. The impaired forelimb had almost completely recovered by the end of the follow-up. In addition, the time needed to remove sticky labels from their impaired forelimb was significantly reduced. However, the spontaneous recovery, complex study design and challenging statistics did not make it possible to discriminate the add-on therapeutic effect. To achieve this, multicenter preclinical trials with greater statistical power will be needed (67).

Cell treatment and housing in EE was started at the same time but it is not known whether this is the most optimal approach. Interestingly, there is recent evidence that a timed sequence of treatments could maximize the therapeutic effects in experimental stroke animals (68). In the case of cell therapy, stabilization with nonselective stimulation such as an enriched environment might be preferable after the initiation of the brain plasticity by the infused cells.

Neuroprotection may also be a prerequisite for delayed cortical plasticity and functional recovery. In the elegant work of Fernández-García et al. (69), transplantation of mesenchymal stem cells alone into stroke mice was not effective as these animals showed permanent sensorimotor deficits in the grid walking test. However, when cells where encapsulated in silk fibroin hydrogel, significant cortical neuroprotection was observed, leading to delayed remapping of forelimb representations and a behavioral recovery similar to that associated with rehabilitation.

### Perilesional Angiogenesis or Gliosis Does not Explain the Behavioral Recovery

The brain repair mechanisms underlying spontaneous or therapy-induced recovery after stroke are still poorly understood (70). However, there is emerging evidence that in particular the perilesional tissue undergoes a major remodeling to foster restitution of function in damaged areas (71).

Angiogenesis, i.e., the formation of new capillaries, is restricted to the border of the infarct and it is claimed to aid in cleansing the necrotic brain tissue (72) as well as providing a site for neuroblast migration (73). MSCs further promote angiogenesis and this has been associated with improved behavioral outcome (74). Consistent with previous studies, we could confirm the ischemia-induced increase of angiogenesis in MCAO rats but were not able to demonstrate any treatment effect. Previous studies that reported increased angiogenesis and improved behavioral performance employed a follow-up period between 14 and 21 days (14, 17, 18). In our study, it may be that ADMSCs only transiently promoted angiogenesis during the early behavioral recovery and this was missed when evaluated after a relatively long follow-up. A temporal monitoring of the extent of angiogenesis at different time points until the end of the follow-up could have been helpful. Furthermore, as previously stated, perhaps a BrdU/RECA-1 double labeling to differentiate old and new blood vessels would have better revealed a treatment effect.

The glial cell response to brain ischemia and glial scar formation are also involved in perilesional remodeling and functional recovery (75). The presence of a glial scar has been claimed to impair repair processes (76). MSCs including ADMSCs decrease perilesional GFAP labeling, a marker for glial cells (17). We detected an increase in the number of glial cells in the perilesional cortex, but no differences between the MCAO groups. In addition, there were no differences in glial scar volume between the MCAO groups. Nonetheless, possible temporal changes in glial cell phenotype attributable to either the ADMSCs and/or the enriched environment cannot be excluded.

### Clinical Implications

Most stroke patients receive some form of rehabilitation. Nonetheless, there is still no agreement about which modalities should be used nor how and when they should be applied. Rehabilitation is a major confounding factor in clinical stem cell trials and it should be taken into account or carefully controlled. Our data strongly support this view. Indeed, the importance of rehabilitation has already been included into the STEPS 3 recommendations (47). However, complex study designs are needed to discriminate add-on and stand-alone therapeutic effects, although these might not be feasible in experimental studies and even less so in clinical trials.

## Conclusions

The combination of multiple regenerative treatments to improve stroke recovery is an attractive strategy. Here we demonstrated that intravenous delivery of ADMSCs and housing in an enriched environment is a safe treatment and improved the behavioral recovery of MCAO rats with a further improvement associated with the combined treatment. The treatment effect was not associated with neuroprotection or altered perilesional angiogenesis or the extent of glial scar formation, but this does not exclude the possibility that there was altered neuronal excitability or axonal sprouting. Further studies with greater statistical power to cope with complex study designs will be needed to determine the optimal protocol and to reveal the true value of combination therapies in stroke.

## Data Availability

All datasets generated for this study are included in the manuscript and/or the Supplementary Files.

## Author Contributions

JM and EO conducted behavioral testing. PK and LC carried out MCAO operations. MJ and SM were responsible for preparation of cells. AB performed histological staining and statistical analysis. MM and SZ were responsible for stereological analysis. AB and JJ drafted the manuscript. All authors approved the final manuscript.

### Conflict of Interest Statement

The authors declare that the research was conducted in the absence of any commercial or financial relationships that could be construed as a potential conflict of interest.
