# Supplementary material for: Combined Adipose Tissue-Derived Mesenchymal Stem Cell Therapy and Rehabilitation in Experimental Stroke
Source: Front Neurol. 2019 Mar 26;10:235. doi: 10.3389/fneur.2019.00235 (PMC6443824; doi:10.3389/fneur.2019.00235)
Supplement: Supplementary file 1 [file Table_1.pdf]

SUPPLEMENTAL TABLE 1 | Body weight (g).

	**Day 0**	**Day 3**	**Day 7**	**Day 21**	**Day 42**
**SHAM+V+S** (n=8)	310.3±16.1	300.6±17.4	314.0±14.6	360.4±18.6	392.4±20.7
**SHAM+C+S** (n=8)	309.3±18.3	297.4±19.3	317.1±20.7	360.4±31.6	392.6±52.8
**SHAM+V+EE** (n=8)	303.5±14.3	294.8±11.5	309.3±12.4	345.3±18.7	379.0±19.6
**SHAM+C+EE** (n=8)	308.6±14.3	301.3±10.6	318.6±9.2	362.4±13.8	398.1±16.6
**MCAO+V+S** (n=12)	312.9±20.6	299.8±19.0	316.8±18.9	362.4±23.8	400.2±37.4
**MCAO+C+S** (n=10)	302.7±16.5	292.4±19.0	307.7±19.1	353.5±22.3	389.8±31.3
**MCAO+V+EE** (n=10)	300.2±13.0	286.6±14.9	303.5±15.3	343.8±21.6	374.4±27.0
**MCAO+C+EE** (n=11)	297.6±6.0	287.3±7.0	299.7±19.2	345.3±15.3	385.1±17.6
**MCAO+V7+EE** (n=8)	308.6±13.7	299.6±11.5	317.9±12.7	364.3±18.8	402.0±21.8
**MCAO+C7+EE** (n=7)	300.1±11.5	294.1±12.8	307.9±13.6	345.9±18.9	374.0±27.2

SHAM=sham-operated; MCAO=middle cerebral artery occlusion; V=2 d vehicle; C=2 d cell infusion; V7=7 d vehicle; C7=7 d cell infusion; S=standard housing; EE=enriched environment.
