# Supplementary material for: Combined Adipose Tissue-Derived Mesenchymal Stem Cell Therapy and Rehabilitation in Experimental Stroke
Source: Front Neurol. 2019 Mar 26;10:235. doi: 10.3389/fneur.2019.00235 (PMC6443824; doi:10.3389/fneur.2019.00235)
Supplement: Supplementary file 2 [file Table_2.pdf]

SUPPLEMENTAL TABLE 2 | Neurological scores.

	Baseline	Day 1	Day 2	Day 3	Day 5	Day 7	Day 21	Day 42
**SHAM+V+S** (n=8)	0±0	0.3±0.5	0.4±0.5	0.6±1.2	0.3±0.7	0.3±0.7	0±0	0±0
**SHAM+C+S** (n=8)	0±0	0±0	0±0	0±0	0±0	0±0	0±0	0±0
**SHAM+V+EE** (n=8)	0±0	0.4±1.1	0±0	0±0	0±0	0±0	0±0	0±0
**SHAM+C+EE** (n=8)	0±0	0.4±1.1	0.4±1.1	0.4±1.1	0.4±1.1	0±0	0±0	0±0
**MCAO+V+S** (n=12)	0±0	1.4±0.9	1.7±1.0	1.6±1.1	1.3±1.1	1.3±1.2	1.2±1.0	0.5±0.9
**MCAO+C+S** (n=10)	0±0	1.5±0.8	1.6±0.8	1.8±0.9	1.7±0.7	1.5±0.8	0.5±0.8	0.6±0.8
**MCAO+V+EE** (n=10)	0±0	1.4±0.7	1.6±0.7	1.2±0.8	1.6±1.0	1.7±0.8	1.3±1.1	0.9±1.0
**MCAO+C+EE** (n=11)	0±0	1.4±0.9	2.0±0.8	1.7±1.0	2.3±1.0	2.0±0.6	1.6±0.7	1.5±0.7
**MCAO+V7+EE** (n=8)	0±0						0.5±0.8	0.8±0.7
**MCAO+C7+EE** (n=7)	0±0						0.9±0.7	1.0±0.8

SHAM=sham-operated; MCAO=middle cerebral artery occlusion; V=2 d vehicle; C=2 d cell infusion; V7=7 d vehicle; C7=7 d cell infusion; S=standard housing; EE=enriched environment.
